# Toxic Effects of p-Chloroaniline on Cells of Fungus *Isaria fumosorosea* SP535 and the Role of Cytochrome P450

**DOI:** 10.3390/toxics13060506

**Published:** 2025-06-16

**Authors:** Shicong Huang, Jiahui Gao, Lin Zhou, Liujian Gao, Mengke Song, Qiaoyun Zeng

**Affiliations:** College of Natural Resources and Environment, South China Agricultural University, Guangzhou 510642, China; huangsc@stu.scau.edu.cn (S.H.); jiahuigao@stu.scau.edu.cn (J.G.); zhoulin@stu.scau.edu.cn (L.Z.); gaoliujian@stu.scau.edu.cn (L.G.)

**Keywords:** p-chloroaniline, fungus, biodegradation, toxicity, cytochrome P450 monooxygenase

## Abstract

Efficient methods to remediate PCA (p-chloroaniline)-polluted environments are urgent due to the widespread persistence and toxicity of PCA in the environment. Microbial degradation presents a promising approach for remediating PCA pollution. However, the PCA-degrading fungi still have yet to be explored. This study confirmed the highly PCA-degrading efficiency of an isolated fungus, *Isaria fumosorosea* SP535. This fungus can achieve a PCA degradation efficiency of 100% under optimal conditions characterized by an initial PCA concentration of 1.0 mM, pH of 7.0 and a temperature of 25 °C. SEM and TEM analyses revealed that the toxicity of PCA resulted in roughened surfaces of *Isaria fumosorosea* SP535 hyphae, voids in the cytoplasm, and thickened cell walls. PCA addition significantly elevated the activities of cytochrome P450 monooxygenase in both cell-free extracts and microsomal fractions in the media, suggesting the important role of the P450 system in PCA metabolization by *Isaria fumosorosea* SP535. The results provide a microbial resource and fundamental knowledge for addressing PCA pollution.

## 1. Introduction

PCA (p-chloroaniline), which is widely used as a chemical raw material in the production of dyes, cosmetics, pesticides, and herbicides, enters the environment through wastewater and waste generated during chemical production processes [1,2,3]. Reports indicate that PCA has been detected in various environments, including water bodies, sediments, agricultural soils, and living organisms [4,5,6]. Due to its toxicity and adverse effects on human health, PCA was classified as a persistent organic pollutant and has been designated as a priority pollutant by the US Environmental Protection Agency (EPA) and European Union legislation [7,8]. PCA has been reported to be carcinogenic and may cause damage to the blood system and nervous system of exposed humans [7,8]. Meanwhile, PCA poses a risk to the safety of marine ecosystems and biological health, ultimately threatening human health through the food chain [9]. Consequently, the development of effective methods for removing PCA residues from the environment has attracted a great deal of attention.

Bioremediation relying on microorganisms is an efficient method for removing organic pollutants in environment [9,10]. Therefore, it is crucial to identify microorganisms with degradation capabilities from the environment. Numerous bacteria have been isolated from various environmental media for the remediation of PCA contamination. *Brevibacillus* S-618, isolated from sludge, was able to completely degrade PCA at a concentration of 180 mg·L^−1^ within 72 h [10]. *Thauera* sp. M9, isolated from soil, completely degraded PCA in culture medium within 30 h [8]. *Bacillus* sp., isolated from textile wastewater, demonstrated a 76% degradation of PCA at a concentration of 100 mg·L^−1^, attaining complete degradation in the presence of lipopeptide surfactant [11]. However, thus far, studies have predominantly focused on bacteria, and PCA-degrading fungi have yet to be reported.

Fungi have been demonstrated to have a remarkable capacity for adapting to harsh environments and have exhibited a strong ability to degrade organic pollutants [12,13]. The degradation efficiency of *Aspergillus* LS-1, isolated from the sludge of a pharmaceutical plant, was 95.41% for 80.14 mg·L^−1^ CTC (chlortetracycline) within 3 d [14]. A yeast strain, *Cutaneotrichosporon dermatis* M503, isolated from the sediment tank of a sewage treatment system at a tetracycline manufacturing facility, achieved a degradation efficiency of 86.62% for TC (tetracycline) within 7 d under optimal conditions [15]. *Aspergillus sydowii* W1, isolated from contaminated soil, demonstrated an 84.05% degradation efficiency for 100 mg·L^−1^ erythromycin at a concentration within 7 d [16]. Eight lignin-degrading fungi capable of degrading polychlorinated biphenyls (PCBs) have been reported, with *Pleurotus ostreatus* removing 98.4% and 99.6% of a PCB mixture in complex and mineral media, respectively, after 6 weeks [17]. Furthermore, some filamentous fungi have been shown to degrade environmental pollutants via cytochrome P450 monooxygenase [18]. With the involvement of cytochrome P450, *Marasmiellus* sp. CBMAI 1062 could nearly completely degrade pyrene (0.08 mg·L^−1^) after 48 h under saline conditions [19]. The degradation efficiency of the herbicide diuron by *Phanerochaete chrysosporium* reached 94% after 10 d cultivation, while the degradation of diuron was inhibited by the addition of 1 mmol/L cytochrome P450 inhibitor ABT (1-aminobenzotriazole) [20]. Sun et al. confirmed that cytochrome P450 is involved in the biodegradation of dichlorvos by *Trichoderma atroviride* T23, and identified five relevant genes: *TaCyp548*, *TaCyp620*, *TaCyp52*, *TaCyp528*, and *TaCyp504* [21]. Therefore, in light of the lack of fungal resources for microbial degradation of PCA, the isolation of fungi with high degradation efficiency for PCA is urgent and necessary for the remediation of PCA pollution.

In this study, the filamentous fungus *Isaria fumosorosea* strain SP535, isolated from soil [22], was selected for experiments on PCA degradation. The objectives of this research were (1) to investigate the toxic effects of PCA on *Isaria fumosorosea* SP535 by assessing biomass, cell morphology, and cellular ultras; (2) to evaluate the degradation efficiency of *Isaria fumosorosea* SP535 on PCA and elucidate the degradation mechanism by analyzing cytochrome P450 monooxygenase activity in free extracts and microsomal fractions; and (3) to examine the influence of cultural factors on PCA degradation by *Isaria fumosorosea* SP535.

## 2. Materials and Methods

### 2.1. Chemicals and Growth Medium

p-chloroaniline (PCA) (CAS# 106-47-8, purity > 99.5%), purchased from Sigma-Aldrich, Chemicals, LTD., St. Louis, MO, USA, was dissolved in sterilized deionized water to obtain a stock solution of 10 mM. The other chemicals and reagents, including K_2_HPO_4_, KH_2_PO_4_, NH_4_Cl, MgSO_4_·7H_2_O, MnSO_4_, and glucose, were supplied by Tianjin Kemiou Chemical Reagent Co., Ltd., Tianjin, China.

Glucose was added into the mineral growth medium according to the modified method of Różalska et al. [23]. The composition of the medium was as follows (g·L^−1^): K_2_HPO_4_ (4.36), KH_2_PO_4_ (1.7), NH_4_Cl (2.1), MgSO_4_·7H_2_O (0.2), MnSO_4_ (0.05), FeSO_4_·7H_2_O (0.01), CaCl_2_·2H_2_O (0.03), glucose (20) and dH_2_O (up to 1000 mL). Integer 25.0 mL was added to each 100 mL Erlenmeyer flask, followed by sterilization at 121 °C for 20 min.

### 2.2. Preparation of Fungal Conidia Inoculum

Fungus, *Isaria fumosorosea* strain SP535, originally isolated from soil and deposited to the collection at Key Laboratory of Biopesticides Innovation and Application of Guangdong Province, South China Agricultural University, Guangzhou, China, was used during these studies. *Isaria fumosorosea* SP535 was incubated on PDA slants for 14 d at 25 °C to allow sufficient spore production. Then, the fungal conidia cultured on PDA slants were harvested with sterilized deionized water containing 0.01% Tween 80 and sieved using filter paper (Whatman No. 2; Science Kit & Boreal Laboratories, New York, NY, USA) into sterile vials. Conidia were counted using a compound microscope and a hemocytometer (0.0625 m^2^; Fuchs-Rosenthal Merck Euro Lab, Darmstadt, Germany) to calibrate a suspension of 1 × 10^8^ conidia mL^−1^.

### 2.3. Determination of PCA Degradation Capacity by Isaria fumonorosea SP535

To determine the degradation capacity of *Isaria fumosorosea* SP535 to PCA, the growth medium prepared as per Section 2.1 was added into 100 mL conical bottle, and the PCA stock solution was added to make the final PCA concentration reach 1.0 mM. The pH was adjusted to 7.0, and 2 mL fungal conidia inoculum prepared as per Section 2.2 was added. Then, the conical bottle was cultured in the constant-temperature shaker at 180 rpm and 25 °C for 120 h. The PCA concentration was measured with 2 mL samples taken every 24 h. The culture samples (2.0 mL) were taken from each treatment at 24 h interval for PCA quantification.

### 2.4. Optimization Studies of Isolated Isaria fumosorosea SP535

Three factors affecting PCA degradation were studied, including initial PCA concentration (0.5, 1.0, 2.0 mM), initial pH (3, 5, 7, 9, 11), and culture temperature (15, 25, 35, 45, 55 °C). Unless otherwise stated, the above experiments were performed at an initial pH of 7.0, an initial PCA concentration of 1.0 mM, and a culture temperature of 25 °C. All cultures were incubated in a thermostatic oscillator for 120 h. PCA concentrations were measured by sampling 2.0 mL solution every 24 h to investigate the influence of culture conditions on the degradation of PCA by *Isaria fumosorosea* SP535.

### 2.5. Estimation of Biomass

To investigate the effects of PCA on *Isaria fumosorosea* SP535 growth, fungal conidia inoculum was inoculated in the medium without PCA and the medium with PCA of 1.0 mM, and cultured at 180 rpm and 25 °C in a constant-temperature shaker for 120 h. Culture samples (2.0 mL) were taken from each treatment every 24 h to measure the biomass of *Isaria fumosorosea* SP535. Total biomass produced by *Isaria fumosorosea* SP535 was quantified by following Ali et al. with some modifications [24]. The whole cultures were filtered through Whatman filter papers (No. 1), which were dried at 80 °C until a constant weight was achieved, and values were expressed as g·L^−1^.

### 2.6. Determination of Cytochrome P450 Monooxygenase

Mycelia obtained after 10 d of culture were converted to spheroplasts by following the method used by Ali et al. [25]. Briefly, 50 mg of mycelia were washed and concentrated 10-fold in 1.0 M sorbitol. The cells were briefly suspended in a mixture solution, including 1.4 M sorbitol, 40 mM HEPES (pH 7.5), 0.5 mM MgCl_2_ and a trace of β-mercaptoethanol. The suspension was shaken for 15 min at 20 °C and then 5 mg·mL^−1^ lyticase was added to lyse the cells. This suspension was shaken for 45 min at 20 °C and then the samples were checked microscopically for the presence of spheroplasts. The spheroplasts were separated from the suspension by centrifugation at 10,000 rpm for 15 min at 4 °C. The spheroplasts from different treatments were suspended in 200 mL fractionation medium with pH at 7.4 (20 mM Tris, 20 mM KH_2_PO_4_, 0.33 M sucrose, 1 mM EDTA, and 0.2% bovine serum albumin). The suspension was homogenized to prevent the aggregation of sub-cellular particles [26,27]. The spheroplast lysate was diluted up to 350 mL with fractionation medium and the pH was adjusted to 7.4. This homogenate was then fractionated by differential centrifugation. Intact spheroplast, nuclei, and large debris were removed by centrifugation at 10,000 rpm for 10 min. The pellet was homogenized for 1 min, diluted, and centrifuged as described above. The supernatant obtained after centrifugation will be referred to as cell-free extract.

The remaining pellet was centrifuged at 20,000 rpm for 30 min; the supernatant was carefully discarded, leaving the mitochondrial peroxisomal fraction. The pellets were resuspended in fractionation medium and centrifuged at 20,000 rpm for 20 min. The supernatant was then decanted, leaving the post-mitochondrial pellet, and centrifuged at 20,000 rpm for 60 min after resuspension in fractionation medium. This centrifugation step resulted in a microsomal pellet which was resuspended in fractionation buffer.

The concentration of functional cytochrome P450 monooxygenase was determined by CO difference spectra [28]. Samples of microsomes containing 1.5 to 2 mg of protein in 1.0 mL of 50 mM Tris-HCl, pH 8.0, in a stopper cuvette were gently sparged with CO for 1 to 2 min, at which time several fine grains of solid sodium dithionite were added. Sparging continued for 1 to 2 min more, and the cuvette was stoppered. Spectra (400 to 500 nm) were recorded at 20 °C using an extinction coefficient of 91 mm^−1^·cm^−1^. The sample was scanned repeatedly; the maximum development of the difference spectrum occurred 5 to 10 min after addition of the sodium dithionite and was recorded.

### 2.7. Sample Preparation for Scanning Electron Microscopy and Transmission Electron Microscopy

To investigate the effects of PCA on the morphology and cell structure of *Isaria fumosorosea* SP535, SEM and TEM were performed on *Isaria fumosorosea* SP535 cultured for 120 h according to Section 2.3.

The culture solution was centrifuged at 5000 rpm for 20 min and supernatant was removed and the fungal mycelia were left. The fungal mycelia were washed thrice with 0.1 M PBS (pH 7.2). The material was then fixed with 2.5% glutaraldehyde in PBS buffer for 3 h at 4 °C then rinsed twice with PBS for 10 min each time followed by rinsing with ddH_2_O. The samples were then placed on glass cover slip (5 × 5 mm) and freeze dried in refrigerator at −80 °C for 3 h followed by overnight drying at 4 °C. The samples were then gold sprayed. The surface morphology of mycelia grown in the presence or absence of PCA was observed with SU8010 (Hitachi, Ltd., Tokyo, Japan) scanning electron microscope (SEM) operated at an accelerated voltage of 5.0 kV.

For transmission electron microscope (TEM), the fungal material, fixed with 2.5% glutaraldehyde in PBS buffer for 3 h at 4 °C as above, was rinsed with same buffer and post-fixed in 1% osmium tetraoxide for 2 h. After dehydration in graded ethanol series, then propylene oxide, the samples were gradually infiltrated and finally embedded in an Epon–Spurr’s resin mixture. An ultrathin section, prepared using Leica CM1950 microtome, were stained with uranyl acetate and lead citrate [29,30] and examined in a JEM 1400 TEM (Hitachi, Ltd., Tokyo, Japan) at 80 kV.

### 2.8. Chemical Analysis

Samples obtained in Section 2.4 were centrifuged at 10,000 rpm for 10 min and supernatant was used for PCA quantification. The PCA concentration in the supernatant was measured by HPLC as described by Hussain et al., with some modifications [31]. HPLC (Shimazdu Co., Ltd., Kyoto, Japan) equipped with a UV detector at 254 nm and a reverse-phase C18 column (250 mm × 4.6 mm) was used. The temperature of the column was maintained at 40 °C, the mobile phase was methanol–water (60:40, *v*/*v*) with a flow efficiency of 0.5 mL·min^−1^. A range of different concentrations of PCA standard were run along with the samples and PCA concentrations were quantified by the PCA standard curve.

## 3. Results and Discussion

### 3.1. The PCA Biodegradation by SP353

**Figure 1:**
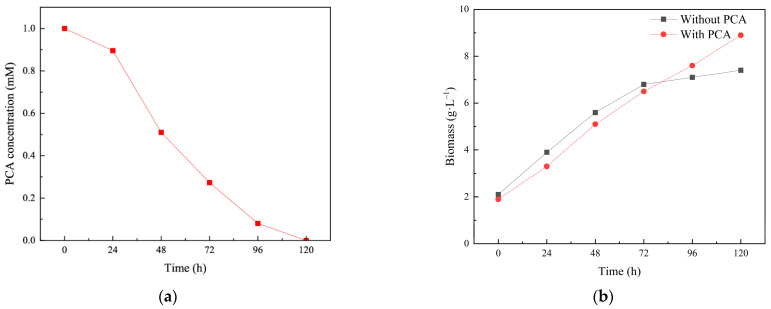
PCA concentration in medium with PCA after inoculation with *Isaria fumosorosea* SP535 within 120 h at 25 °C, pH 7.0 (**a**) and biomass of *Isaria fumosorosea* SP535 incubated in the medium with/without PCA (**b**) within 120 h at 25 °C, pH 7.0.

The PCA degradation capability of *Isaria fumosorosea* SP535 was presented in Figure 1a. Figure 1b showed the influence of PCA on the growth of this strain. During the first 72 h, the biomass of *Isaria fumosorosea* SP535 in the media containing PCA was lower than that in the media without PCA, with values of 6.8 g·L^−1^ and 6.5 g·L^−1^, respectively. However, at 96 h, the biomass of *Isaria fumosorosea* SP535 in the media containing PCA surpassed that in the media without PCA, with values of 7.6 g·L^−1^ and 7.1 g·L^−1^, respectively. After 120 h, the biomass of *Isaria fumosorosea* SP535 in the two media reached 7.4 g·L^−1^ (without PCA) and 8.9 g·L^−1^ (with PCA), respectively. This observation suggested that *Isaria fumosorosea* SP535 may utilize PCA as a carbon source to enhance its growth in the later stages of culture.

Over the past few decades, several PCA-degrading bacteria have been isolated and utilized in studies focused on PCA contamination remediation. For instance, a novel strain, *Delftia tsuruhatensis* H1, isolated by Zhang et al., demonstrated the ability to completely degrade PCA at a concentration of 400 mg·L^−1^ within 25 h [32]. *Acinetobacter baylyi* strain GFJ2 has been employed for the degradation of aniline and halogenated aniline, achieving a 97% reduction in PCA at a concentration of 0.2 mM within 72 h [33]. *Thauera* sp. M9, isolated from contaminated soil by Kumar et al., exhibited a degradation efficiency of 100% for PCA at a concentration of 300 mg·L^−1^ in 30 h, alongside significant degradation capacity for 2-CA (2-Chloroaniline), 3-CA (3-Chloroaniline), and 3,4-DCA (3, 4-Dichloroaniline) [8]. *Brevibacillus* S-618, isolated from effluent by Li et al., accomplished complete degradation of PCA at a concentration of 180 mg·L^−1^ at a temperature of 30 °C, pH 7, and an air–water ratio of 0.3 m^3^/m^3^·min within 72 h [10]. *Bacillus* sp., isolated by Carolin et al., 2021, was able to completely degrade PCA at an initial concentration of 100 mg·L^−1^ within 72 h in the presence of a lipopeptide surfactant [11]. However, fungi capable of degrading PCA have not been reported previously. In this study, *Isaria fumosorosea* strain SP535, a filamentous fungus isolated from soil, successfully degraded PCA at a concentration of 1.0 mM under pH 7.0 and a temperature of 25 °C within 120 h. To the best of our knowledge, this study represents the first instance of isolated PCA-degrading fungi, thereby enriching the biological resources available for PCA biodegradation.

### 3.2. Effect of PCA on the Growth and Cellular Structure of Isaria fumosorosea SP535

**Figure 2:**
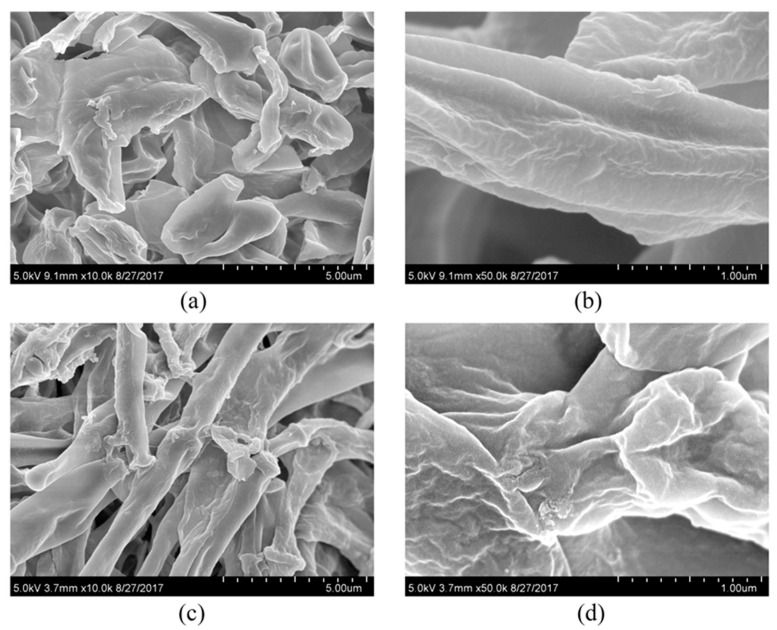
SEM images of *Isaria fumosorosea* SP535 after 120 h of incubation: (**a**,**b**) SEM images of *Isaria fumosorosea* SP535 incubated for 120 h in medium without PCA; (**c**,**d**) SEM images of *Isaria fumosorosea* SP535 incubated for 120 h in medium containing PCA. All images were observed using a SU8010 (Hitachi) scanning electron microscope (SEM) operating at an acceleration voltage of 5.0 kV.

**Figure 3:**
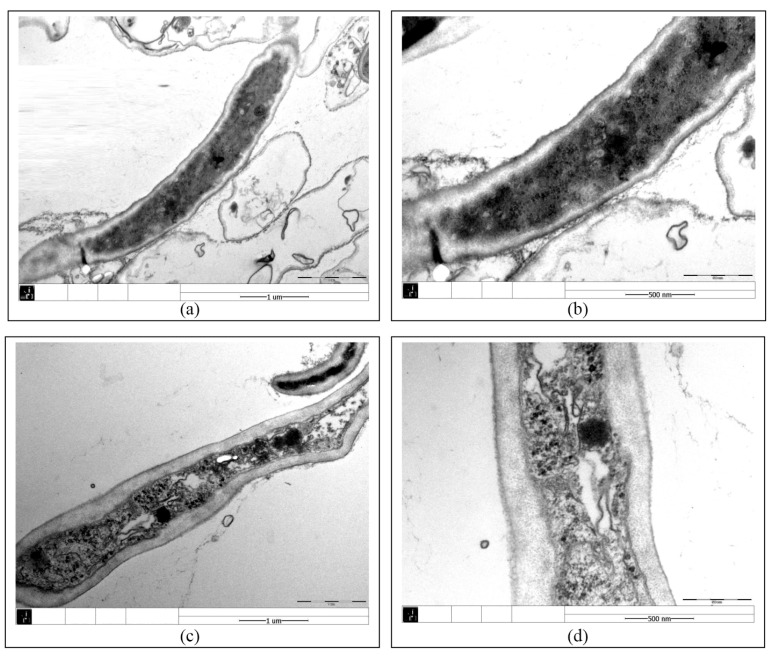
TEM images of *Isaria fumosorosea* SP535 after 120 h of incubation: (**a**,**b**) TEM images of *Isaria fumosorosea* SP535 incubated for 120 h in medium without PCA; (**c**,**d**) TEM images of *Isaria fumosorosea* SP535 incubated for 120 h in medium containing PCA. All images were observed using JEM 1400 TEM at an accelerated voltage of 80 kV.

Figure 2 and Figure 3 showed the effect of PCA on the hyphal morphology and cell ultrastructure of *Isaria fumosorosea* SP535 during biodegradation. In a batch culture without PCA, the outer surface morphology of *Isaria fumosorosea* SP535 was smooth and clean (Figure 2a,b), while the surface of fungal mycelia growing in the presence of PCA was rough and the deposition of PCA on the outer surface of mycelia was clear (Figure 2c,d). At the same time, the fungal mycelia in PCA-treated samples had obvious space in the cytoplasm and the cell wall was thickened (Figure 3).

The toxicity of environmental pollutants usually leads to changes in the cellular structure of microorganisms. The toxicity of alkyl phenols caused similar morphological changes in the cells of *Aspergillus tubingensis*, with tight mycelium arrangement resulting in rough surface and some oval-shaped vacuoles in the cytoplasm [34]. Similarly, when exposed to tributyltin, the protoplasts of fungal cells were destroyed and some gaps appeared between the cell membrane and the cell wall [35]. *Phanerochaete chrysosporium* exposed to 100 mg·L^−1^ PFOS (perfluorooctane sulfonate) resulted in the formation of a large number of intracellular cavities, which was attributed to the fact that PFOS can bind to membrane phospholipids, thus preventing the process of membrane synthesis and leading to the formation of cavities [36]. The increase in fungal cell wall thickness may be a response to adverse environmental conditions. *Wallemia* can adapt to high-salinity environments by significantly increasing cell wall thickness [37]. Cell wall thickness increased significantly in *Metarhizium robertsii* cultured in the presence of nonylphenol [30]. In this study, the toxicity of PCA caused the mycelium surface of *Isaria fumosorosea* SP535 to become rough, the cytoplasm showed obvious gaps, and the cell wall thickened.

### 3.3. Factors Affecting PCA Degradation by SP353

#### 3.3.1. Effects of Initial PCA Concentrations on Biodegradation

**Figure 4:**
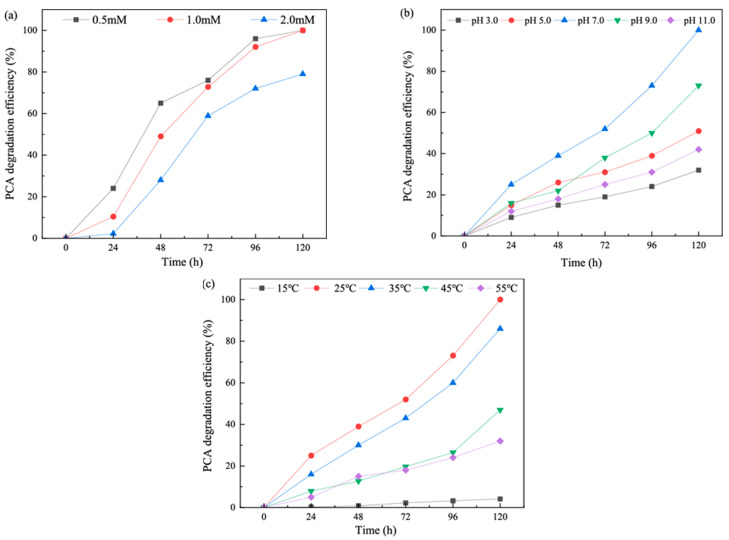
Degradation efficiency (%) of PCA in the medium after incubating *Isaria fumosorosea* SP535 for 120 h incubation at different degradation conditions: (**a**) different PCA initial concentration, (**b**) different pH and (**c**) different temperature.

The effects of different initial concentrations of PCA on biodegradation were shown in Figure 4a. The PCA biodegradation by *Isaria fumosorosea* SP535 decreased with the increase in PCA concentration within the experimental concentration range. When the initial concentration of PCA was 0.5 mM (6.38 mg·L^−1^) and 1.0 mM (12.76 mg·L^−1^), the degradation efficiency of PCA by *Isaria fumosorosea* SP535 reached 100% within 120 h. When the initial concentration of PCA was further increased to 2.0 mM (25.51 mg·L^−1^), the degradation efficiency of PCA by *Isaria fumosorosea* SP535 decreased to 79%. This indicated that *Isaria fumosorosea* SP535 can effectively degrade PCA at a concentration below 1.0 mM.

Since the toxicity of PCA may inhibit the growth of degrading bacteria and even lead to the death of degrading bacteria, the concentration of PCA will affect the degradation effect of degrading bacteria on PCA. The PCA-degrading bacteria *Bacillus* sp. had a degradation efficiency of 100% for PCA with a concentration lower than 100 mg·L^−1^, but the degradation efficiency of PCA dropped to less than 85% when the concentration of PCA increased to 150 mg·L^−1^ [11]. After 30 h culture, when the concentration of PCA in the medium was 300 mg/L, the OD_600_ of *Thauera* sp. M9 increased from 0.1 to 0.372 and the degradation efficiency was 100%, while when the concentration of PCA in the medium was increased to 400 mg·L^−1^ and 500 mg·L^−1^, the OD_600_ increased from 0.1 to 0.321 and 0.265, and the degradation efficiencies were 28% and 15%, respectively [8]. Similarly, Vangnai and Petchkroh reported that the growth of *Acinetobacter baumannii* CA2, *Pseudomonas putida* CA16 and *Klebsiella* sp. CA17 was almost completely inhibited when the concentration of PCA in the medium was increased from 0.2 mM to 1.6 mM [38]. When the PCA concentration increased from 180 mg·L^−1^ to 270 mg·L^−1^, the growth of *Brevibacillus* S-618 was inhibited, the cell dry weight decreased from 1.6 g·L^−1^ to 0.25 g·L^−1^, and the degradation efficiency of PCA decreased from 86.7% to 9% [10].

#### 3.3.2. Effects of pH on Biodegradation

The effects of different pH values on biodegradation are shown in Figure 4b. As shown in Figure 4b, the PCA degradation efficiency by *Isaria fumosorosea* SP535 increased with the increase in pH in the acidic environment, while the degradation efficiency of PCA by *Isaria fumosorosea* SP535 decreased with the increase in pH in the alkaline environment. The optimal pH for PCA degradation by *Isaria fumosorosea* SP535 was 7, and the degradation efficiencies at 96 h and 120 h PCA were 73% and 100%, respectively. At initial pH values of 3 and 11, the PCA degradation efficiencies were 32% and 42%, indicating that *Isaria fumosorosea* SP535 was more adapted to an alkaline environment.

pH affected the microbial growth and the enzymatic activity of the catabolic system [14,39], thereby impacting the biodegradation of PCA. *Brevibacillus* S-618, isolated by Li et al., exhibited the highest PCA degradation efficiency of 86.3% at pH 7; however, its degradation efficiency decreased when the pH was either increased or decreased [10]. Similarly, Carolin et al. found that *Bacillus* sp. demonstrated optimal PCA degradation at pH 7 [11]. *Bacillus licheniformis* strain ycsd02, isolated by Ding et al., could degrade 94% of 20 mg·L^−1^ 4-CA after 100 h of culture at pH 7.0, with degradation efficiency dropping to 25% at pH 9.0 [40]. *Acinetobacter baylyi* strain GFJ2 can completely degrade 25 mg·L^−1^ PCA at pH 7 [33]. Additionally, the optimal pH for the cytochrome P450 enzyme produced by *Isaria fumosorosea* ranges from 5.7 to 7.0, facilitating PCA biodegradation by *Isaria fumosorosea* SP535 [25]. Furthermore, pH affects the bioavailability of certain pesticides, influencing their absorption and degradation by microorganisms. For example, the bioavailability of the piscicide TFM (3-trifluoromethyl-4-nitrophenol) increases at lower pH levels, making it more easily absorbed by organisms [41]. The bioavailability of four neonicotinoids including imidacloprid, acetamiprid, clothianidin, and thiamethoxam, was positively correlated with soil pH, with higher bioavailability noted at elevated pH levels [42].

#### 3.3.3. Effects of Temperature on Biodegradation

The PCA degradation efficiencies by *Isaria fumosorosea* SP535 at different temperatures were shown in Figure 4c. When the temperature increased from 15 °C to 25 °C, the degradation efficiency of PCA by *Isaria fumosorosea* SP535 increased, while the degradation efficiency of PCA by *Isaria fumosorosea* SP535 decreased when the temperature increased from 25 °C to 55 °C. When the temperature was 15 °C, the degradation efficiency of PCA by *Isaria fumosorosea* SP535 was 4%, and *Isaria fumosorosea* SP535 hardly degraded PCA at low temperature. At a temperature of 25 °C, *Isaria fumosorosea* SP535 achieved complete degradation of PCA within 120 h. With the further increase in temperature 35, 45 and 55 °C, the degradation efficiencies of PCA by *Isaria fumosorosea* SP535 were 86%, 47% and 32%, respectively.

The effects of temperature on PCA biodegradation in previous studies indicated an initial increase followed by a decrease, likely due to the inhibitory effects of both high and low temperatures on strain growth and enzyme activity. *Brevibacillus* S-618 showed the highest degradation efficiency of PCA (85.9%) at an incubation temperature of 30 °C, and the dry cell weight of the strain was more than 1.5 g·L^−1^, whereas the growth of *Brevibacillus* S-618 was inhibited at incubation temperatures of 22 °C and 38 °C, and the growth efficiency of *Brevibacillus* S 618 was significantly lower than 0.75 g·L^−1^, resulting in PCA degradation efficiencies of less than 40% in both cases [10]. Ding et al. reported similar findings, indicating that the optimal degradation temperature for the *Bacillus licheniformis* strain ycsd02 was 30~32 °C, with rapid declines in degradation efficiency at temperatures above or below this range [40]. Additionally, *Deltia tsuruhatensis* H1 was capable of completely degrading PCA at a concentration of 300 mg·L^−1^ at 30 °C [32]. The degradation efficiency of *Thauera* sp. M9 for 300 mg·L^−1^ PCA reached 99.29% at 30 °C, while at 25 °C, 35 °C, 40 °C, and 45 °C, the efficiencies were only 30%, 22.58%, 2.92%, and 2.39%, respectively [8]. Furthermore, Wang et al. demonstrated that high temperatures (39 °C) inhibited the expression of cytochrome P450 family 11 subfamilies A member 1, revealing the possibility that a high temperature might inhibit the expression of cytochrome P450 and thus inhibit the biodegradation of PCA by *Isaria fumosorosea* SP535 [43].

### 3.4. Potential Mechanism of PCA Degradation by Isaria fumosorosea SP535

Cytochrome P450 enzymes were investigated in this study to elucidate the potential degradation mechanism of PCA by the isolated fungus. As heme monooxygenases that are widely present in filamentous fungi, cytochrome P450 enzymes were believed to be involved in the transformation of organic matter performed by these organisms [44,45,46]. The role of the cytochrome P450 system in PCA biodegradation was assessed by measuring the cytochrome P450 monooxygenase activity in the cell-free extracts and microsomal components derived from fungal mycelia cultivated in media with and without PCA. The activities of cytochrome P450 monooxygenase in the cell-free extract and microsomal fraction were enhanced by the addition of PCA, exhibiting concentrations of 0.02 and 0.03 nmol·mg^−1^ protein in the control group, and 0.31 and 0.84 nmol·mg^−1^ protein in the PCA treatment group, respectively (Table 1). This finding suggested that the cytochrome P450 system may contribute to PCA degradation by SP353.

Similarly, Al-Hawash et al. reported that the expression of oxidation-related cytochrome P450 genes increased from 0.94-fold to 5.45-fold under n-hexadecane (HXD) conditions, indicating that HXD stimulates cytochrome P450 production [47]. Furthermore, the expression of P450 in the HBCD (hexabromocyclododecane) degrading bacterium *Rhodopseudomonas palustris* increased five-fold after 12 h of treatment with HBCD at 35 °C [48]. Wu et al. documented that cytochrome P450 monooxygenase activity rose from 0 to nearly 10 U·mg^−1^ following the inoculation of *Rhodopseudomonas marshes* in soil contaminated with the herbicide butachlor, and the timing of *EthB* regulatory gene expression correlated with butachlor degradation, suggesting that butachlor induces *EthB* gene expression to synthesize cytochrome P450 monooxygenase, thereby facilitating the degradation of butachlor by *Rhodopseudomonas marshes* [49]. In this study, the increased activity of cytochrome P450 monooxygase in the PCA treatment group suggested that *Isaria fumosorosea* SP535 may degrade PCA by initiating the cytochrome P450 system.

## 4. Conclusions

This study reported an isolate of filamentous fungus *Isaria fumosorosea* SP535 with high PCA-degrading ability for the first time. The optimal degradation parameters were an initial PCA concentration of 1.0 mM; initial pH of 7.0; and growth temperature of 25 °C. The ability of *Isaria fumosorosea* SP535 to degrade PCA displays its potential use in the remediation of PCA-contaminated sites. Fungal cells grown on PCA showed high cytochrome P450 enzymes activities, suggesting that *Isaria fumosorosea* SP535 may metabolize PCA through the P450 system.

However, there are still some problems with the application of *Isaria fumosorosea* SP535 for PCA degradation. PCA may co-exist with a variety of pollutants in the actual environment, and the survivability of *Isaria fumosorosea* SP535 in the actual environment needs to be further confirmed. The degradation processes in this study were carried out in the laboratory, and the remediation effect of *Isaria fumosorosea* SP535 on PCA pollution in the actual environment is not yet known. Some strains, when applied to the environment, may affect the community composition and structure of the indigenous microorganisms in the environment, which in turn may affect the environmental ecology. The above issues should be further investigated in future studies.

## Figures and Tables

**Table 1:** Activities (nmol·mg^−1^) of cytochrome P450 monooxygenase in cell-free extract and microsomal fraction of *Isaria fumosorosea* SP535 incubated for 120 h in the medium without PCA and containing PCA.

Treatment	Cell-Free Extract	Microsomal Fraction
Control	0.02 ± 0.00 ^b^	0.03 ± 0.01 ^b^
PCA	0.31 ± 0.02 ^a^	0.84 ± 0.04 ^a^

All data were analyzed using one-way ANOVA. Values are means (±S.E) of three independent replicates. Different letters for data in the same column represent significant differences (*p* < 0.05).

## Data Availability

The data are available on request.
